# Can Different Forest Structures Lead to Different Levels of Therapeutic Effects? A Systematic Review and Meta-Analysis

**DOI:** 10.3390/healthcare9111427

**Published:** 2021-10-23

**Authors:** Eunsoo Kim, Sujin Park, Soojin Kim, Yeji Choi, Junghee Cho, Sung-il Cho, Hae-ryoung Chun, Geonwoo Kim

**Affiliations:** 1 Future Forest Strategy Department, Forest Human Service Division, National Institute of Forest Science, Seoul 02455, Korea; euncarp2@gmail.com (E.K.); snowshoe@korea.kr (S.P.); kimsoojinsj@korea.kr (S.K.); usmile.choi@gmail.com (Y.C.); jjh6758@korea.kr (J.C.); 2 Department of Public Health Sciences, Graduate School of Public Health, Seoul National University, Seoul 08826, Korea; persontime@hotmail.com (S.-i.C.); mamimihae@gmail.com (H.-r.C.); 3 Institute of Health and Environment, Seoul National University, Seoul 08826, Korea

**Keywords:** stand density, canopy density, affective restoration, cognitive restoration, physiological relaxation, subgroup analysis

## Abstract

In recent decades, forests have expanded from natural resources for conservation and production to health-promoting resources. With the growing body of evidence supporting the therapeutic effects of forests, the number of investigations on the relationship between forest characteristics and therapeutic effects have increased. However, quantitative synthesis of primary studies has rarely been conducted due to a limited number of health studies including forest description and high heterogeneity of forest variables. In this study, we conducted a systematic review and meta-analysis to evaluate the relationship between the forest structure and the therapeutic effect. We systematically searched the studies examining the therapeutic effects of forests with different stand density and canopy density and synthesized the results. As a result of subgroup analysis, we found that stand density modifies the therapeutic effects. Emotional and cognitive restoration showed greatest improvement in low-density forests with a stand density of less than 500/ha and the therapeutic effects diminish as the stand density increases. The impact of canopy density was not found due to a lack of studies reporting canopy density. Although some limitations remain, the findings in this study have great significance in providing the basis for establishing management strategies of forests for therapeutic use.

## 1. Introduction

Human use of nature to promote mental and physical health has a long history and continues accumulating plenty of empirical evidence. In the 1980s, two representative theoretical frameworks were proposed that explained how experiencing nature benefits human health. One is the attention restoration theory (ART) which suggests cognitive recovery through experience in nature. According to ART, natural environments are rich in components aiding recovery from mental fatigue [1,2]. Exposure to nature evokes involuntary attention, rather than voluntary attention that causes directed attention fatigue, result in the restoration of directed attention, giving more opportunities to restore cognitive abilities [1,2,3,4]. The other one is the stress reduction theory (SRT) that emphasizes emotional recovery and stress reduction through aesthetic experience in nature [5,6]. Viewing natural scenery arouses positive emotions, limits negative thoughts, and alters the autonomic nervous system activity towards being parasympathetic-dominant, leading to more relaxed states [5,6,7,8]. In addition to these two approaches, several health-promoting pathways in the natural environment have been proposed, such as promoting physical activity and social contact, providing a quality environment for improving mental health [9,10]. Plenty of studies have accumulated, leading to recognition of the therapeutic potential of nature. Before 2007, however, most investigations did not distinguish types of the natural environment because studies have focused primarily on demonstrating the health-promoting effects of natural exposure and its mechanisms [11].

Since 2007, some East Asian and European countries have started to utilize urban forests, peri-urban forests, and conservation areas to improve public health and well-being [11,12,13,14,15,16,17]. Accordingly, research on forests, a specific natural environment, has been increasing. Several clinical trials conducted walking, staying, and exercising sessions in the forest and examined the therapeutic effect of forests. They demonstrate that forest have effects on reducing anxiety and depression [18,19,20,21], reducing stress [8,22,23,24], promoting physiological relaxation [25,26,27,28,29,30], improving cognitive function [31,32,33], relieving inflammation [34,35,36,37], improving immune function [37,38,39,40,41], and preventing cardiovasculars [35,36,42,43]. Overall, these studies demonstrate that individuals gain health benefits through mental and physical interactions with forest elements. The forest environments consist of scenery, scent, sounds, phytoncides, anions, sunlight, microclimate environments, and topography. These elements act as therapeutic factors by stimulating the five senses [1,17,28,44,45,46,47], promoting psychological and physiological relaxation [25,26,27,28,29,30,48], supporting cognitive recovery [49], providing beneficial chemicals to the human body [50,51,52,53,54,55,56,57], and giving a recreational space [33,58].

Recently, studies have started to examine the relationship between therapeutic outcomes and various forest variables. Investigations were conducted on a wide range of forest variables such as spatial structure [59,60,61], openness-enclosure [59,62,63], vegetation density [59,64,65], tree cover density [66], stand structure [67], species composition [44], management [68,69], and physical environment [45,70,71,72]. Those investigations aimed to assess and estimate the benefits of the forest by its characteristics, determine adequate levels of forest variables, and provide guidance to create and manage forest sites for therapeutic use [73]. Furthermore, one systematic review examined the differences in health effects according to the forest variables. However, few studies included forest variables at the time of the review, and there was a limit to elucidating the relationship because the forest variables investigated in each study were different. We recognized that more articles have been reported since the publication of the review, and that the search needs to be narrowed down to specific forest variables. Therefore, we conducted a systematic review and a meta-analysis to update more recent evidence and identify the relationship between the therapeutic effects and the structural variables of forests: stand density and canopy density.

The structure of the forest affects the sense of openness, lightness, comfort, and aesthetics that humans feel, so it can be expected that the structural characteristics will change the therapeutic effect of the forest. Further, we focused on the density of trees, a major component of the forest environment. Previous studies revealed that high-density trees limit visual access and ease of movement, and may disturb immersion in nature in that it threatens a sense of security [64,74,75]. Conversely, a forest setting with a low vegetation density can give a sense of openness but it can create a boring and monotonous landscape [66,76,77,78,79]. In this regard, we considered stand density—a number of trees per hectare— and canopy density—the top density of trees—as a major factor in modifying the therapeutic impacts of forest sites. Besides, we decided to include stand density and canopy density for this review because these are measurable variables widely used in the forestry sector and can be changed by short-term forest management.

A systematic review is a research method that summarizes the latest empirical evidence relevant to a specific research question, and meta-analysis is a statistical method that quantitatively synthesizes the data from searched studies. In general, meta-analysis is performed to estimate the effect size more accurately by synthesizing homogeneous studies. It can also be conducted to confirm whether study-level variables change the outcomes of each study [80]. The key challenge of this study was to investigate the moderating role of stand density and canopy density. Therefore, we systemically summarized previous studies reporting both therapeutic effect and structural variables of the forest. Then we performed a meta-analysis to investigate whether the structural variables of the forest modify the therapeutic effect. We expected this study to contribute to developing strategies to increase the therapeutic use of forests.

## 2. Materials and Methods

We followed the guidelines of the Cochrane Handbook for Systematic Reviews of Interventions (Version 6.2) [80], and we identified and evaluated the available literature in accordance with the Preferred Reporting Items for Systematic Reviews and Meta-Analyses (PRISMA) checklist [81]. The study was registered in OSF database under the number DOI:10.17605/OSF.IO/D7J5Q

### 2.1. PICO and Eligibility Criteria

In order to address a clearly formulated research question, we set our research question by specifying the population, intervention, comparison, outcome (PICO). We also established eligibility criteria following the PICOS framework for the study selection, displayed in Table 1, as follows:

- P: All human participants (both healthy and unhealthy).
- I: Spending time in the forest or urban forest with the description of forest environment in terms of stand density and canopy density—stand density, tree density, trees/ha, basal area, canopy density, canopy openness, canopy closure, sky view factor etc.
- C: Studies with a control site or not.
- O: The studies that contained quantitative outcomes related to cognitive restoration, psychological restoration, physiological relaxation, and stress reduction.

### 2.2. Search Strategy

We took 24 pilot searches and finalized search keywords to gather relevant studies and obtain reproducible search results. The search keywords contain keywords relevant to forest-based activities, forest variables, and therapeutic effects (Table 2). We conducted a systematic literature search of four databases PubMed, EBSCO, Web of Science, and Scopus. We searched published articles in English from the inception year to the end of June 2020.

### 2.3. Study Selection Process

**Figure 1:**
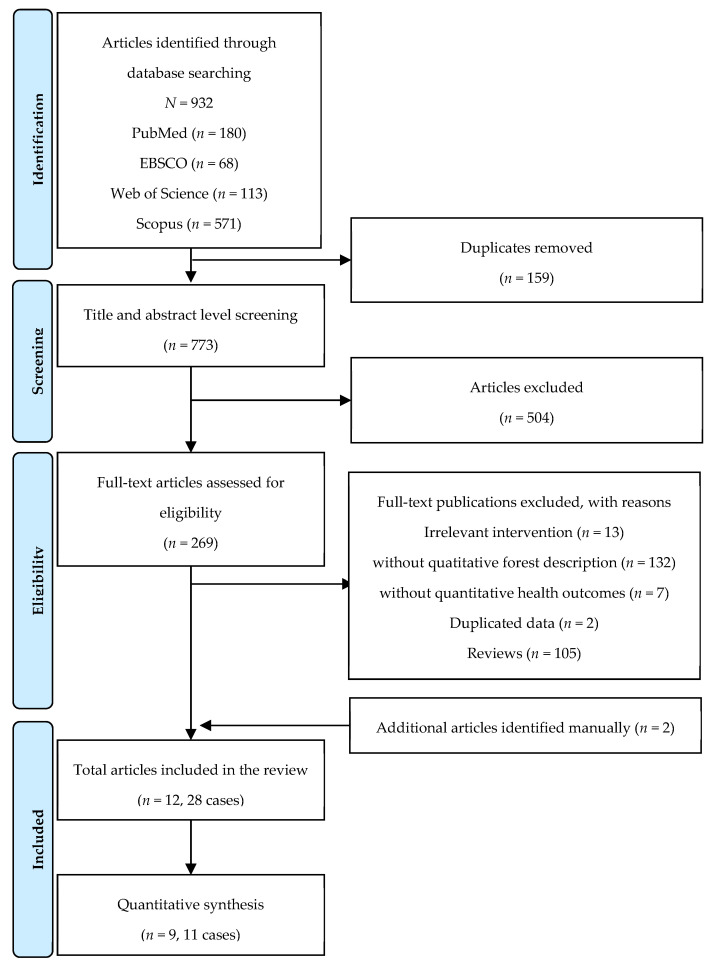
Flow diagram illustrating the selection process.

From the database search, we found a total of 932 studies with 180 on PubMed, 68 on EBSCO, 113 on Web of Science, and 571 on Scopus. The results were exported to EndNote Citation Manager software (Endnote X9.3.3, Clarivate Analytics, London, UK). After removing 159 duplicates, the titles and abstracts of the 773 publications were reviewed. After removing 504 explicitly irrelevant cases, two investigators independently reviewed the full text for 269 studies based on eligibility criteria (E.K. and Y.C.). Disagreements during the screening process were resolved by two other investigators (G.K. and S.P.). We included ten studies from our database search and added two studies that were manually identified. Thus, in total, 12 studies were included in the review (Figure 1).

### 2.4. Data Extraction

Two investigators (E.K. and S.K.) independently used the same data extraction form and cross-checked them. Data were extracted from individual studies to identify studies, determine the homogeneity between studies, and estimate the effect size of individual studies. The extracted data included study information, samples, forest characteristics, environmental characteristics, intervention, and outcome measurements as follows.

- Study information: year of publication, author, country, city, study design, conducted date, time of measurement
- Samples: sample size, gender, and age
- Forest characteristics: longitude, latitude, altitude, stand density (trees/ha), canopy density (%), diameter at breast height (cm), height (m), dominant tree species
- Environmental characteristics: temperature (°C), relative humidity (%), radiant heat
- (°C), wind speed (m/s), illuminance (lx), noise level (dB)
- Intervention: activities, activity duration, and frequency
- Outcome: measurement indices, pre-measurement (M ± SD), post-measurement (M ± SD), change in measurement (M ± SD), inter-trial correlation

In the case of stand density reported as basal area (m^2^/ha) and growing stock (m^3^/ha), we converted it into the number of trees per hectare. In the case of canopy density reported as canopy openness, we reversed and unified it.

### 2.5. Methodological Quality

To collect all relevant studies reporting forest structural variables, we did not impose strict restrictions on study design and included non-randomized studies in the review. ROBINS-I was used to evaluate the study’s methodological quality, according to the recommendations of the Cochrane Handbook [80]. ROBINS-I is a useful methodological assessment tool in a systematic literature review that includes non-randomization studies [82]. ROBINS-I evaluates seven areas of bias at the stage of pre-intervention, at intervention, and post-intervention. Two investigators (E.K. and G.K.) independently assessed the risk of bias by answering the signaling questions for each bias area. The degree of bias is derived as low risk of bias, moderate risk of bias, serious risk of bias, critical risk of bias, and no bias information. If the evaluation result is a low risk of bias, it can be regarded as a study similar to a well-performed randomized controlled trial. In the case of a moderate risk of bias, it provides fair evidence but is difficult to compare with a randomized controlled trial. In the case of a serious risk of bias, there is a significant defect in at least one area, but it is difficult to regard the overall quality as low. Critical risk of bias means that there are many problems to provide useful evidence and cannot be used to synthesize research results.

### 2.6. Statistical Analysis

We conducted the statistical analysis using R 4.04 and R Studio with the “metafor” and other R packages. We extracted effect estimates from individual studies, estimates overall effect, conducted subgroup analysis and sensitivity analysis.

#### 2.6.1. Extracting Estimates of Effect

Since the control group for each study was different, pre-and post-measurement of individual forest environments were extracted and used for analysis. We calculated standardized mean differences (SMD) to estimate the effect size. The mean, standard deviation, number of samples, and inter-trial correlation of pre-and post-measured values were used for SMD calculation. If the study did not report the inter-trial correlation, we estimated inter-trial correlation according to the recommendations of the Cochrane Handbook [80]. When several forest environments were investigated in one study, we extracted the effect size separately for each forest environment. As Cohens et al. [83] suggested, we interpreted the result of SMD 0.20–0.49 as “small” effect size, 0.50–0.79 as “medium” effect size, and ≥0.80 as “large” effect size.

#### 2.6.2. Estimating Overall Effect Size

There are two statistical models for meta-analysis. One is a fixed-effect model, which assumes that studies share a common effect size and that differences in results result from sampling error. The other is a random-effects model, which assumes that the true effect size varies by study and is distributed around the overall mean. As we hypothesized that the therapeutic effect of forest varies by forest structure, we used a random-effects model to estimate effect size.

#### 2.6.3. Sub-Group Analysis

We conducted a subgroup analysis to investigate the relationship between the structural variable of forest sites and their therapeutic effect. We divided structural variables into three or four subgroups based on their homogeneity and calculated the proper sample size using G*Power 3.1. software to ensure a satisfactory power value. To assess inter-study heterogeneity in subgroup analysis, we used Cochrane’s Q test (*p* < 0.10 for statistical significance) and I^2^ (I^2^ > 50% used as a threshold for significant heterogeneity). We observed whether the two structural variables affected the healing effect by the significance test of subgroup differences and the comparison of heterogeneity according to the subgroup analysis. We also report the 95% confidence interval of SMD for each subgroup.

#### 2.6.4. Sensitivity Analysis

Finally, we took a sensitivity analysis to check whether individual studies affect the effect estimation. The leave-one-out method was used for sensitivity analysis, and we assessed the degree of change through Rstudent, diffits, Cook’s D, covratio, T^2^, Q_resid_, hat value, and DFBETAS values (the results are available in the Supplementary Materials Figure S1).

## 3. Results

### 3.1. Study Characteristics

A total of 12 studies were included in this review and characteristics of those are shown in Table 3. Of the 12 included studies, one was a randomized controlled trial, seven were randomized crossover studies, and four were non-randomized studies. All studies were conducted in forest settings, and eight studies conducted interventions in multiple forest sites. Studies were conducted in seven countries, including Japan (four studies; nine sites), China (three studies; nine sites), Korea (one study, one site), Taiwan (one study, one site), Finland (one study; four sites), Poland (one study, one site), Spain (one study, one site). Thus, a total of 26 different forest sites were examined. The main intervention methods were staying (seven studies), walking (four studies), or both (one study). The duration of interventions in most studies was within 120 min (11 studies), and one study conducted a 180 min-length intervention.

A total of 685 participants were included in 12 studies, and the number of samples ranged from 8 to 346. The average age of participants in the included study ranged from 20.8 to 60.0 years. Four studies included only young adults, one study included only middle-aged adults, six included both young and middle-aged, and one included both middle-aged and elderly.

The therapeutic effects were reported as emotional restoration, cognitive restoration, and physiological relaxation. Emotional restoration included improvement in mood states such as anxiety (eight studies; eighteen sites), depression (seven studies; sixteen sites), anger (seven studies; sixteen sites), confusion (six studies; fifteen sites), fatigue (six studies; fifteen sites), vigor (seven studies; nineteen sites), negative and positive affect (four studies; nine sites). Cognitive restoration included restorative experience (five studies; thirteen sites). Physiological relaxation included lowering blood pressure (four studies; seven sites), heart rate and pulse rate (four studies; seven sites), relaxation effects measured through heart rate variability and stress hormone (one study; two sites). Additionally, one study measured the blood concentration of monoterpenes as an indicator of therapeutic effect.

Prior to the meta-analysis, we reviewed participant characteristics, species compositions, microclimate, noise, and the timing of measurement to rule out possible confounding factors. As a result, nine studies of twelve included studies were used for meta-analysis. Since thermal discomfort can disrupt the therapeutic experience, we excluded three studies that are difficult to be considered homogeneous in terms of environmental characteristics (temperature and humidity). There were some differences in how each forest was managed. However, it was not thought that there would be a significant difference in the perception of the forest environment among the participants, mainly non-experts, as most of the studies investigated conifer-dominant mixed forest.

### 3.2. Methodological Quality

Methodological quality was assessed using ROBINS-I. Overall, the risk of bias was moderate in most studies (Table 4). At the pre-intervention stage, low confounding bias was found in most studies, with the exception of two studies that did not provide information on potential confounding factors. Selection bias was mainly low, however moderate selection bias was found in four studies due to lack of randomization and control groups. At the intervention stage, all studies found low classification bias due to clearly defined forest sites, activities. At the post-intervention stage, most of the studies found low bias by deviations and missing data, with the exception of one study that did not provide sufficient information. Moderate bias in measurement found in most studies reflects the lack of blinding in studies using self-reported measures. Most studies were concerned that there was a moderate bias in their reporting due to the lack of evidence to justify the analytical methods, such as pre-registered protocol. One study was assessed as having serious reporting bias due to incompletely reported results.

### 3.3. Stand Density as an Effect Modifier of Therapeutic Effect

We classified stand density into four groups: low for <500/ha, medium for 500–1000/ha, high for 1000–1500/ha, and very high for ≥1500/ha. In the case of emotional restoration, improving anxiety, depression, anger, frustration, and fatigue, enhancing vitality, alleviating negative emotions, and promoting positive emotions were reported. The restorative outcome scale (ROS), which measures restorative experience, was reported regarding cognitive restoration. For physiological relaxation, lowering effects on blood pressure, pulse rate, and heart rate were reported. The results for each outcome indices are described in subsections.

#### 3.3.1. Emotional Restoration

##### Anxiety

**Figure 2:**
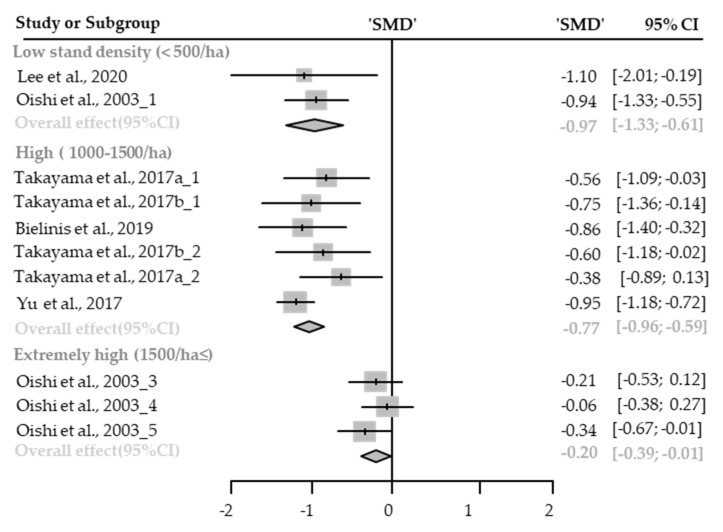
Three forest plots of the change in anxiety in subgroup analysis by stand density: low (<500/ha), high (1000–1500/ha), extremely high (1500/ha≤). The data are reported as standardized mean differences (SMD) and 95% confidence intervals (CIs). The diamond at the bottom presents the overall effect. The plotted squares denote SMD, and the whiskers denote their 95% CIs.

Twelve cases with different stand densities were used for meta-analysis. Anxiety state significantly relieved in the forest environment (SMD −0.52; 95% CI −0.76 to −0.28; *p* < 0.0001; I^2^ = 76%; 106 participants). As a result of subgroup analysis, there was a significant difference in the effect size according to the stand density (P_subgroup_ < 0.0001; Figure 2). “Big” effect in low-density environments (SMD −0.97; 95% CI −1.33 to −0.61; *p* < 0.0001; I^2^ = 0%), and “medium” effect in high-density environments (SMD −0.77; 95)% CI −0.96 to −0.59; *p* < 0.0001; I^2^ = 9%) “small” effect in extremely high-density environments (SMD −0.20; 95% CI −0.39 to −0.01; *p* = 0.0389; I^2^ = 0%) appeared. One case in an environment with moderate stand density was excluded from the subgroup analysis.

##### Depression

**Figure 3:**
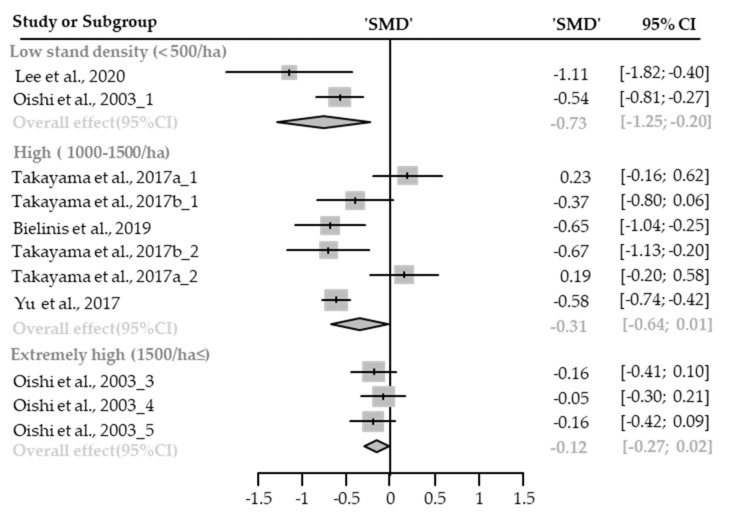
Three forest plots of the change in depression in subgroup analysis by stand density: low (<500/ha), high (1000–1500/ha), and extremely high (1500/ha≤). The data are reported as standardized mean differences (SMD) and 95% confidence intervals (CIs). The diamond at the bottom presents the overall effect. The plotted squares denote SMD, and the whiskers denote their 95% CIs.

Twelve cases with different stand densities were used for meta-analysis. Depression state significantly relieved in the forest environment (SMD −0.29; 95% CI −0.47 to −0.10; *p* = 0.028; I^2^ = 78%; 106 participants). As a result of subgroup analysis, there was a marginally significant difference in the effect size according to the stand density (P_subgroup_ = 0.0778; Figure 3). “Medium” effect in low-density (SMD −0.73; 95% CI −1.25 to −0.20; *p* = 0.0067; I^2^ = 54%) appeared, however, no significant effect was shown in high-density environments (SMD −0.31; 95% CI −0.64 to 0.01; *p* = 0.0594; I^2^ = 81%) and extremely high-density environments (SMD −0.12; 95% CI −0.27 to 0.02; *p* = 0.1034; I^2^ = 0%). One case in an environment with moderate stand density was excluded from the subgroup analysis.

##### Anger

**Figure 4:**
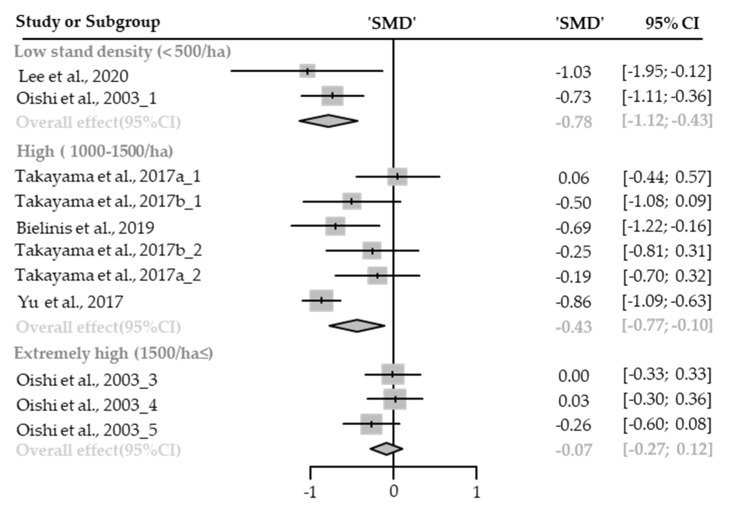
Three forest plots of the change in anger in subgroup analysis by stand density: low (<500/ha), high (1000–1500/ha), and extremely high (1500/ha≤). The data are reported as standardized mean differences (SMD) and 95% confidence intervals (CIs). The diamond at the bottom presents the overall effect. The plotted squares denote SMD, and the whiskers denote their 95% CIs.

Twelve cases with different stand densities were used for meta-analysis. Anger state significantly relieved in the forest environment (SMD −0.35; 95% CI −0.58 to −0.12; *p* = 0.0026; I^2^ = 73%; 106 participants). As a result of subgroup analysis, there was a significant difference in the effect size according to the stand density (P_subgroup_ = 0.0032; Figure 4). A “medium” effect in low-density (SMD −0.78; 95% CI −1.12 to −0.43; *p* < 0.0001; I^2^ = 0%), and a “small” effect in high-density environments (SMD −0.43; 95% CI −0.77 to −0.10; *p* = 0.01; I^2^ = 68%) appeared. While there was no significance effect shown in extremely high-density environments (SMD −0.07; 95% CI −0.27 to 0.12; *p* = 0.4469; I^2^ = 0%). One case in an environment with moderate stand density was excluded from the subgroup analysis.

##### Confusion

**Figure 5:**
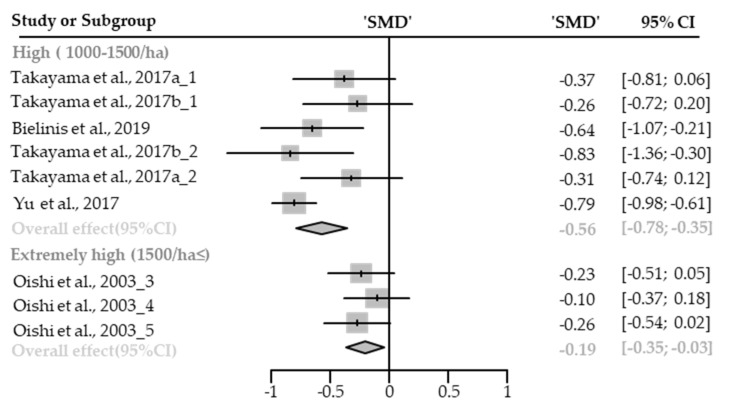
Two forest plots of the change in confusion in subgroup analysis by stand density: high (1000–1500/ha), extremely high (1500/ha≤). The data are reported as standardized mean differences (SMD) and 95% confidence intervals (CIs). The diamond at the bottom presents the overall effect. The plotted squares denote SMD, and the whiskers denote their 95% CIs.

Eleven cases with different stand densities were used for meta-analysis. Confusion state was significantly relieved in the forest environment (SMD −0.41; 95% CI −0.60 to −0.23; *p* < 0.001; I^2^ = 71%; 226 participants). As a result of subgroup analysis, there was a significant difference in the effect size according to the stand density (P_subgroup_ = 0.0020; Figure 5). “Medium” effect in high-density (SMD −0.56; 95% CI −0.78 to −0.35; *p* < 0.0001; I^2^ = 47%), and “small” effect in extremely high-density environments (SMD −0.19; 95% CI −0.35 to −0.03; *p* = 0.0181; I^2^ = 0%) appeared. One case in the low- and moderate-density environment was excluded from the subgroup analysis.

##### Fatigue

**Figure 6:**
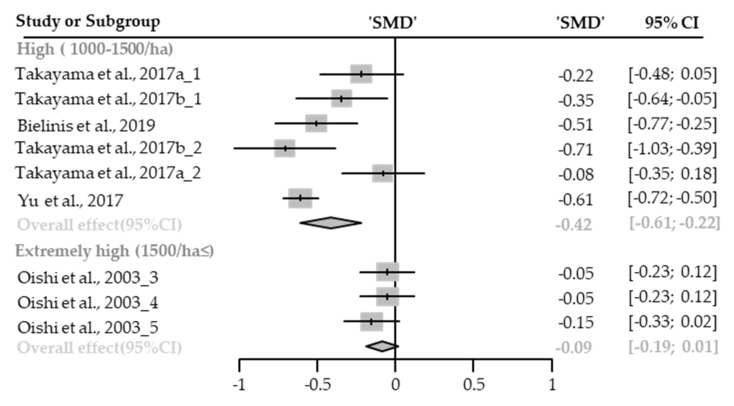
Two forest plots of the change in fatigue in subgroup analysis by stand density: high (1000–1500/ha), and extremely high (1500/ha≤). The data are reported as standardized mean differences (SMD) and 95% confidence intervals (CIs). The diamond at the bottom presents the overall effect. The plotted squares denote SMD, and the whiskers denote their 95% CIs.

Eleven cases with different stand densities were used for meta-analysis. Fatigue state significantly relieved in the forest environment (SMD −0.29; 95% CI −0.45 to −0.12; *p* = 0.0006; I^2^ = 87%; 226 participants). As a result of subgroup analysis, there was a significant difference in the effect size according to the stand density (P_subgroup_ < 0.0001; Figure 6). A “small” effect in high-density (SMD −0.42; 95% CI −0.61 to −0.22; *p* < 0.0001; I^2^ = 75%), and a very “small” effect in extremely high-density environments (SMD −0.09; 95% CI −0.19 to −0.01; *p* = 0.0890; I^2^ = 0%) appeared. One case in the low- and moderate-density environment was excluded from the subgroup analysis.

##### Vigor

**Figure 7:**
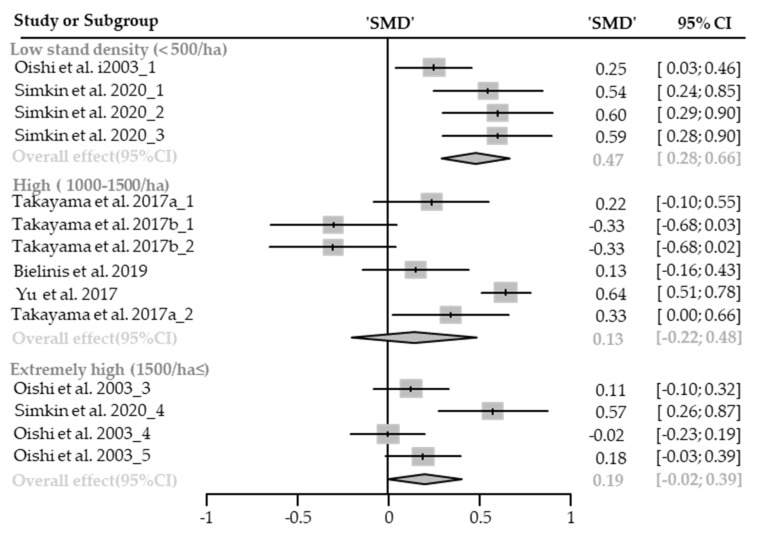
Three forest plots of the change in vigor in subgroup analysis by stand density: low (<500/ha), high (1000–1500/ha), extremely high (1500/ha≤). The data are reported as standardized mean differences (SMD) and 95% confidence intervals (CIs). The diamond at the bottom presents the overall effect. The plotted squares denote SMD, and the whiskers denote their 95% CIs.

Fifteen cases with different stand densities were used for meta-analysis. Vigor significantly improved in the forest environment (SMD 0.23; 95% CI 0.07 to 0.39; *p* = 0.0041; I^2^ = 84%; 300 participants). As a result of subgroup analysis, there was a significant difference in the effect size according to the stand density (P_subgroup_ = 0.0053; Figure 7). A “small” effect in low-density (SMD 0.47; 95% CI 0.28 to 0.66; *p* = 0.0002; I^2^ = 55%) appeared, however, there were no significant effects shown in high-density environments (SMD 0.13; 95% CI −0.22 to 0.48; *p* = 0.4787; I^2^ = 90%) and extremely high-density environments (SMD 0.19; 95% CI −0.02 to 0.39; *p* = 0.0821; I^2^ = 70%). One case in an environment with moderate stand density was excluded from the subgroup analysis.

##### Negative Affect

**Figure 8:**
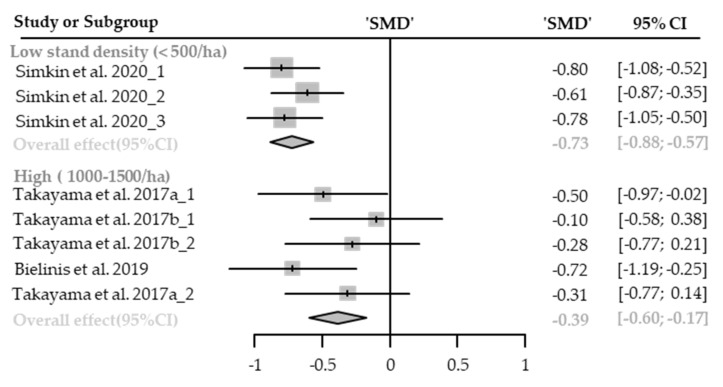
Two forest plots of the change in negative affect in subgroup analysis by stand density: low (<500/ha), high (1000–1500/ha). The data are reported as standardized mean differences (SMD) and 95% confidence intervals (CIs). The diamond at the bottom presents the overall effect. The plotted squares denote SMD, and the whiskers denote their 95% CIs.

Nine cases with different stand densities were used for meta-analysis. Negative affect significantly alleviated in the forest environment (SMD −0.56; 95% CI −0.70 to −0.42; *p* < 0.0001; I^2^ = 34%; 120 participants). As a result of subgroup analysis, there was a significant difference in the effect size according to the stand density (P_subgroup_ = 0.0282; Figure 8). A “medium” effect in low-density (SMD −0.73; 95% CI −0.88 to −0.57; *p* < 0.0001; I^2^ = 0%), and a “small” effect in high-density (SMD −0.39; 95% CI −0.60 to −0.17; *p* = 0.0004; I^2^ = 0%) appeared, one case in an environment with extremely high stand density was excluded from the subgroup analysis, and no case with moderate stand density.

##### Positive Affect

**Figure 9:**
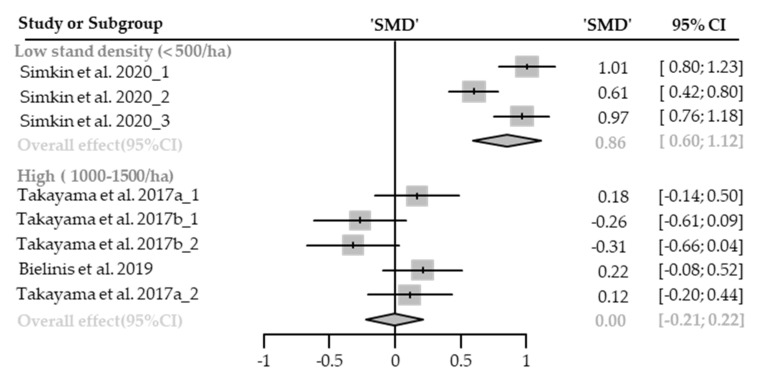
Two forest plots of the change in positive affect in subgroup analysis by stand density: low (<500/ha), high (1000–1500/ha). The data are reported as standardized mean differences (SMD) and 95% confidence intervals (CIs). The diamond at the bottom presents the overall effect. The plotted squares denote SMD, and the whiskers denote their 95% CIs.

Nine cases with different stand densities were used for meta-analysis. Positive affect significantly improved in the forest environment (SMD 0.34; 95% CI 0.05 to 0.63; *p* = 0.0202; I^2^ = 92%; 120 participants). As a result of subgroup analysis, there was a significant difference in the effect size according to the stand density (P_subgroup_ < 0.0001; Figure 9). A “big” effect in low-density (SMD 0.86; 95% CI 0.60 to 1.12; *p* < 0.0001; I^2^ = 80%) appeared, however, no significant effect was shown in high-density environments (SMD 0.00; 95% CI −0.21 to 0.22; *p* = 0.9824; I^2^ = 55%). One case in an environment with extremely high stand density was excluded from the subgroup analysis, and no cases with moderate stand density were excluded.

### 3.3.2. Cognitive Restoration

#### Restorative Experience

**Figure 10:**
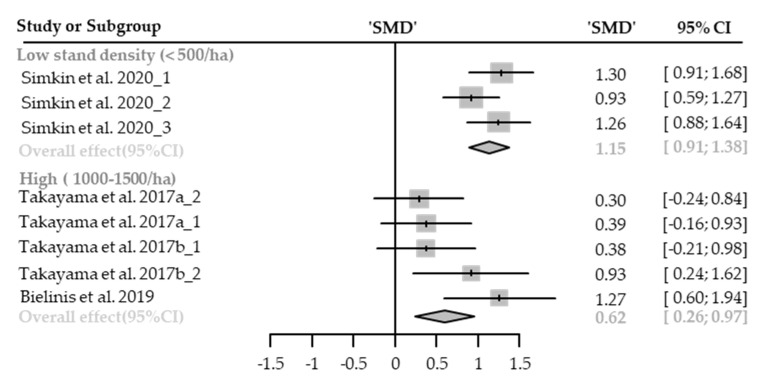
Two forest plots of the change in ROS in subgroup analysis by stand density: low (<500/ha), high (1000–1500/ha). The data are reported as standardized mean differences (SMD) and 95% confidence intervals (CIs). The diamond at the bottom presents the overall effect. The plotted squares denote SMD, and the whiskers denote their 95% CIs.

Nine cases with different stand densities were used for meta-analysis. Restorative experience measured by ROS significantly increased in the forest environment (SMD 0.87; 95% CI 0.62 to 1.11; *p* < 0.0001; I^2^ = 60%; 120 participants). As a result of subgroup analysis, there was a significant difference in the effect size according to the stand density (P_subgroup_ = 0.0385; Figure 10). A “big” effect in low-density (SMD 1.15; 95% CI 0.91 to 1.38; *p* < 0.0001; I^2^ = 20%), and a “medium” effect in high-density (SMD 0.62; 95% CI 0.26 to 0.97; *p* = 0.0007; I^2^ = 43%) appeared. One case in an environment with extremely high stand density was excluded from the subgroup analysis, and no cases with moderate stand density were excluded.

### 3.3.3. Physiological Relaxation

#### Diastolic Blood Pressure

**Figure 11:**
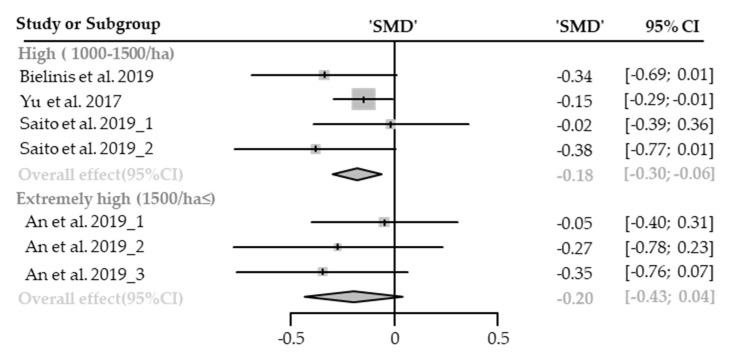
Two forest plots of the change in DBP in subgroup analysis by stand density: high (1000–1500/ha), extremely high (1500/ha≤). The data are reported as standardized mean differences (SMD) and 95% confidence intervals (CIs). The diamond at the bottom presents the overall effect. The plotted squares denote SMD, and the whiskers denote their 95% CIs.

Seven cases with different stand densities were used for meta-analysis. Diastolic blood pressure significantly lowered in the forest environment (SMD −0.18; 95% CI −0.29 to −0.08; *p* = 0.0008; I^2^ = 0%; 179 participants). As a result of subgroup analysis, there was no significant difference in the effect size according to the stand density (P_subgroup_ = 0.9027; Figure 11). A “small” effect appeared both in high-density (SMD −0.18; 95% CI −0.30 to −0.06; *p* = 0.0031; I^2^ = 0%) and extremely high-density environments (SMD −0.20; 95% CI −0.43 to −0.04; *p* = 0.53; I^2^ = 0%). No cases with low- and moderate-density environments were reported.

#### Systolic Blood Pressure

**Figure 12:**
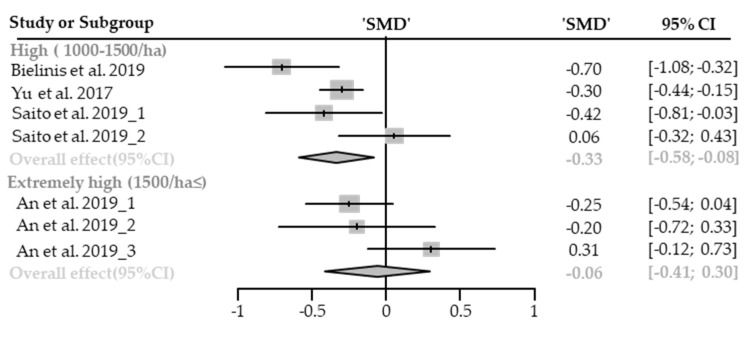
Two forest plots of the change in SBP in subgroup analysis by stand density: high (1000–1500/ha), extremely high (1500/ha≤). The data are reported as standardized mean differences (SMD) and 95% confidence intervals (CIs). The diamond at the bottom presents the overall effect. The plotted squares denote SMD, and the whiskers denote their 95% CIs.

Seven cases with different stand densities were used for meta-analysis. Systolic blood pressure significantly lowered in the forest environment (SMD −0.23; 95% CI −0.43 to −0.03; *p* = 0.0249; I^2^ = 61%; 179 participants). As a result of subgroup analysis, there was no significant difference in the effect size according to the stand density (P_subgroup_ = 0.2179; Figure 12). A “small” effect appeared in high-density environments (SMD −0.33; 95% CI −0.58 to −0.08; *p* = 0.05; I^2^ = 63%), however, no significant effect was shown in extremely high-density environments (SMD −0.06; 95% CI −0.41 to 0.30; *p* = 0.7459; I^2^ = 56%). No cases with low- and moderate-density environments were reported.

#### Heart Rate or Pulse Rate

**Figure 13:**
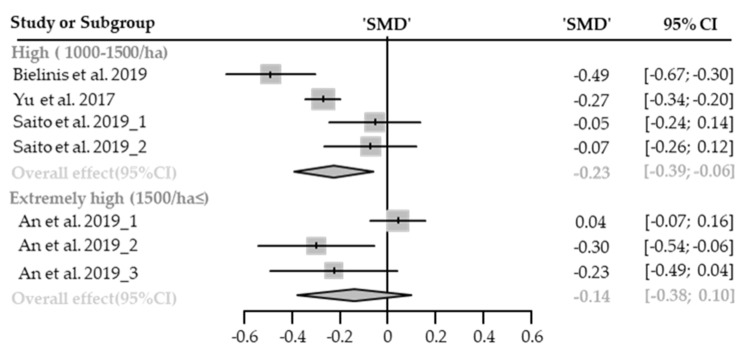
Two forest plots of the change in heart rate and pulse rate in subgroup analysis by stand density: High (1000–1500/ha), extremely high (1500/ha≤). The data are reported as standardized mean differences (SMD) and 95% confidence intervals (CIs). The diamond at the bottom presents the overall effect. The plotted squares denote SMD, and the whiskers denote their 95% CIs.

Seven cases with different stand densities were used for meta-analysis. Heart rate or pulse rate significantly lowered in the forest environment (SMD −0.19; 95% CI −0.33 to −0.05; *p* = 0.0091; I^2^ = 83%; 179 participants). As a result of subgroup analysis, there was no significant difference in the effect size according to the stand density (P_subgroup_ = 0.5565; Figure 13). A “small” effect appeared in high-density environments (SMD −0.23; 95% CI −0.39 to −0.06; *p* = 0.0076; I^2^ = 79%), however, no significant effect was shown in extremely high-density environments (SMD −0.14; 95% CI −0.38 to 0.10; *p* = 0.2528; I^2^ = 75%). No cases with low- and moderate-density environments were reported.

### 3.4. Canopy Density as an Effect Modifier of Therapeutic Effect

The canopy density was classified into three groups: low for <50%, medium for 50–70%, and high for ≥70%. We conducted subgroup analysis on blood pressure, heart rate, and pulse rate, which are indicators of physiological relaxation effect. Due to the limited number of studies, we cannot conduct subgroup analysis on emotional and cognitive restoration. The results for each effect indicator are described in subsections.

#### 3.4.1. Physiological Relaxation

##### Diastolic Blood Pressure

**Figure 14:**
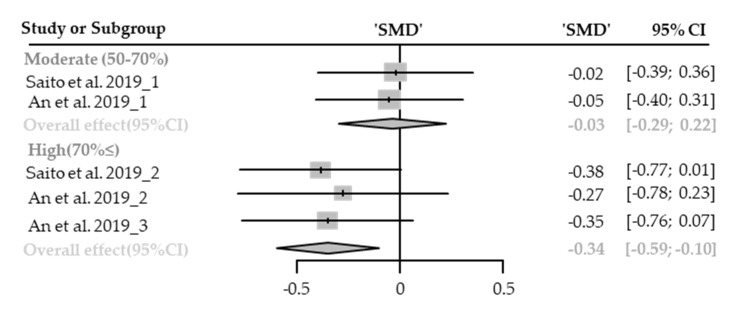
Two forest plots of the change in DBP in subgroup analysis by canopy density: moderate (50–70%), extremely high (70%≤). The data are reported as standardized mean differences (SMD) and 95% confidence intervals (CIs). The diamond at the bottom presents the overall effect. The plotted squares denote SMD, and the whiskers denote their 95% CIs.

Five cases with different canopy densities were used for meta-analysis. Diastolic blood pressure significantly lowered in the forest (SMD −0.19; 95% CI −0.37 to −0.02; *p* = 0.0328; I^2^ = 0%; 30 participants). As a result of subgroup analysis, there was a marginally significant difference in the effect size according to the canopy density (P_subgroup_ = 0.0890; Figure 14). A “small” effect appeared in high-density environments (SMD −0.34; 95% CI −0.59 to −0.10; *p* = 0.0066; I^2^ = 0%) while no significant effect was shown in moderate-density environments (SMD −0.03; 95% CI −0.29 to 0.22; *p* = 0.8005; I^2^ = 0%). No cases with low canopy densities were reported.

##### Systolic Blood Pressure

**Figure 15:**
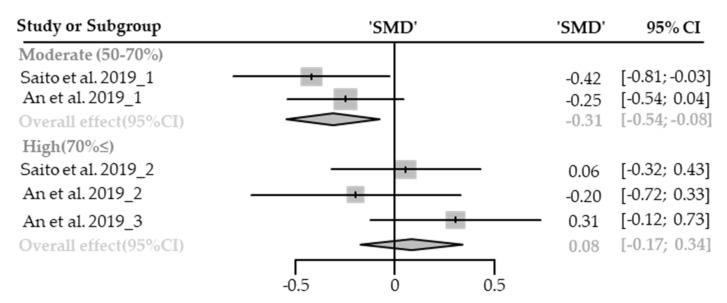
Two forest plots of the change in SBP in subgroup analysis by canopy density: moderate (50–70%), extremely high (70%≤). The data are reported as standardized mean differences (SMD) and 95% confidence intervals (CIs). The diamond at the bottom presents the overall effect. The plotted squares denote SMD, and the whiskers denote their 95% CIs.

Five cases with different canopy densities were used for meta-analysis. Systolic blood pressure significantly lowered in the forest (SMD −0.11; 95% CI −0.35 to 0.13; *p* = 0.3744; I^2^ = 48%; 30 participants). As a result of subgroup analysis, there was a significant difference in the effect size according to the canopy density (P_subgroup_ = 0.00271; Figure 15). A “small” effect appeared in moderate-density environments (SMD −0.31; 95% CI −0.35 to 0.13; *p* = 0.0096; I^2^ = 0%) while no significance effect was shown in high-density environments (SMD 0.08; 95% CI −0.17 to 0.34; *p* = 0.5288; I^2^ = 6%). No cases with low canopy densities were reported.

##### Heart Rate or Pulse Rate

**Figure 16:**
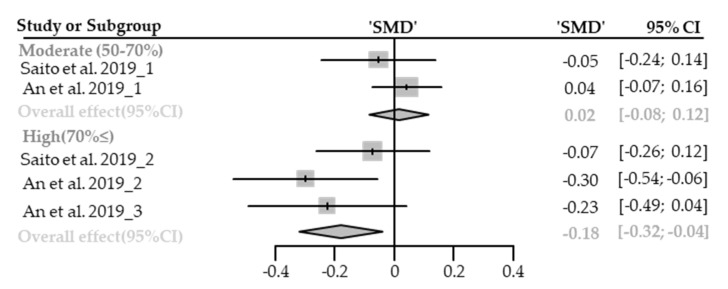
Two forest plots of the change in heart rate and pulse rate in subgroup analysis by canopy density: moderate (50–70%), extremely high (70%≤). The data are reported as standardized mean differences (SMD) and 95% confidence intervals (CIs). The diamond at the bottom presents the overall effect. The plotted squares denote SMD, and the whiskers denote their 95% CIs.

Five cases with different canopy densities were used for meta-analysis. There was no significant change in heart rate or pulse in the forest environment (SMD −0.09; 95% CI −0.21 to 0.03; *p* = 0.1460; I^2^ = 51%; 30 participants). As a result of subgroup analysis, there was a significant difference in the effect size according to the canopy density (P_subgroup_ = 0.0260; Figure 16). A “small” effect was appeared in high-density environments (SMD −0.18; 95% CI −0.32 to −0.04; *p* = 0.0126; I^2^ = 11%) while no significance effect was shown in moderate-density environments (SMD 0.02; 95% CI −0.08 to 0.12; *p* = 0.7409; I^2^ = 0%). No cases with low canopy densities were reported.

### 3.5. Other Possible Effect Modifier

**Figure 17:**
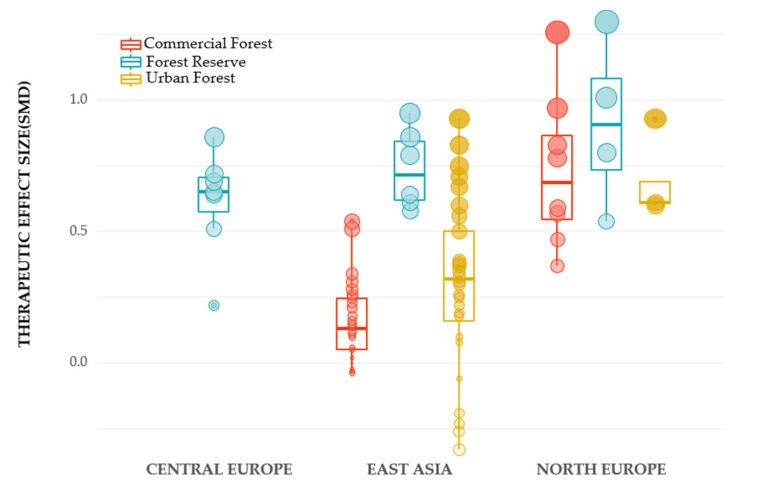
Box plots comparing the therapeutic effects by regions (Central Europe, East Asia, and North Europe) and forest types (commercial forest, forest reserve, urban forest). The overlapped dots present standardized mean differences (SMD) on emotional restoration and cognitive restoration extracted from included studies.

The included studies were conducted on forest sites in East Asia, Central Europe, and Northern Europe (Figure 17). We also classified management types of the forest into “commercial forest,” a managed forest for timber production and commercial use, “forest reserve,” a strictly protected forest, and “urban forests” forest located within walking distance from the city. In addition to the structural characteristics, we found region and management type as a possible effect-modifier. We compared the therapeutic effects on mental health, including emotional and cognitive restoration, by region and management type. By region, the biggest effect was shown in Northern Europe (mean 0.912, median 0.905), followed by East Asia (mean 0.738, median 0.715), and Central Europe (mean 0.612, median 0.650). By management type, the effect size showed the biggest in a forest reserve in the order of Northern Europe (mean 0.912; median 0.905), East Asia (mean 0.738; median 0.715), and Central Europe (mean 0.612; median 0.650). By management type and region in East Asia, forest reserve (mean 0.738; median 0.715) had the biggest effect size, followed by urban forest (mean 0.292; median 0.320) and commercial forest (mean 0.151; median 0.130). On the other hand, in the case of Northern Europe, forest reserve (mean 0.913; median 0.905) has the biggest effect size, followed by commercial forest (mean 0.730; median 0.685) and urban forest (mean 0.688; median 0.610).

## 4. Discussion

In recent years, the therapeutic potential of forests has been gradually recognized in various countries, and the number of investigations of the therapeutic effect according to the forest characteristics has increased. In this regard, we tried to identify the relationship between therapeutic effects and forest structure. We hypothesized that stand density and canopy density will act as major effect-modifiers affecting forests’ therapeutic effects on emotional restoration, cognitive restoration, and physiological relaxation. This study systematically searched, summarized, and synthesized previous studies examining the therapeutic effects of various forest environments with different structural variables.

As a result of meta-analysis, stand density significantly modified the effect on emotional and cognitive restoration. Anxiety, anger, and negative affect decreased the most in the low-density forest where the stand density was less than 500/ha. In contrast, the effect size became smaller or insignificant as the tree density increased. The relieving effect on confusion and fatigue also decreased as stand density increase and showed the smallest effect size in the extremely high tree density of 1500/ha or higher. The improvement of depression and enhancement of vitality and positive effects were significant only in the low-density forest with a stand density less than 500/ha. There was the largest increase in restorative experience in the low-density forest with a stand density less than 500 trees per hectare. Hence, mental recovery was greatest in low-density forests and decreased as the stand density of trees increased. On the other hand, there was no significant difference in physiological relaxation by stand density. It might be due to physiological outcomes were investigated only under high or extremely high stand density conditions.

By dividing the subgroups, we observed a decrease in heterogeneity within each subgroup and found significant differences between the subgroups. It suggests that stand density modifies the therapeutic effect of forests. However, in depression, anger, fatigue, and vitality, we found non-negligible heterogeneity in forest environments with high stand density between 1000/ha and 1500/ha. High heterogeneity in those subgroups suggests a need for more tightly divided subgroups with a stand density greater than 1000 trees per hectare. Furthermore, few studies investigated moderate-density forests with a stand density between 500/ha and 1000/ha. Considering that previous studies estimated the most significant therapeutic effect in the middle level of stand density [59,60,65], it is necessary to investigate the therapeutic effect of moderate-density forests with a stand density between 500/ha and 1000/ha.

Depending on canopy density, the physiological relaxation indices showed inconsistent and opposite trends. This is thought to be due to the limited number of investigations reporting the canopy density. A review of literature on the therapeutic effects of forests noticed the insufficient number of studies that measured and reported canopy density of forest sites. Furthermore, most literature reported tree cover, which indicates a level of greenness at the landscape level, not at the spatial level that humans can perceive. For future guidance on the therapeutic use of forests, studies should include detailed descriptions of the forest sites where the participants perform their activities.

The structural variables used in this study—stand density and canopy density—are widely reported variables in forest management, growth monitoring, and tree ecology studies. Through a review of publications from 1989 to 2021 reporting the health effects of forests, we found that structural characteristics of forest environments began to be described earnestly after 2017. This may reflect the international trends of increasing collaboration between forestry and the health sector [13,95,96]. Forests have long been treated as natural resources for production or conservation. However, in recent decades, several countries in East Asia, Europe, and North America have begun to recognize forests’ preventive medical potential and managed forests to promote public health [11,95,96,97]. For example, Japan has continuously certified forests rich in therapeutic effects through on-site verification since 2006 [98]. Korea has been creating national healing forests since 2010 [95,99]. Denmark established the Healing Forest Garden Nacadia [100] and Finland established the first ‘Forest Wellness Trail’ in 2010. The German state of Mecklenburg-Pomerania also started operating a legal designation system for forests with excellent recreational and therapeutic properties in 2019 [99]. In addition, Finland, North America, and Canada have recently supported health promotion activities based on natural environments [24,97].

This study has several limitations. First, the study design was not limited to randomized controlled trials (RCTs). Forest therapy has been studied in earnest over the past decade, and quantitative forest descriptions have only recently begun to be included in intervention information in clinical trials. Most RCTs were excluded from the review process as the information on the forest environment was insufficient. For this reason, we included not only RCTs but also randomized cross-over studies and NRCTs as the scope of this study. Secondly, we extracted effect size using pre-and post- measurements of each forest site rather than differences in forest sites and controls. The studies used in the meta-analysis did not contain the control group examining pre-and post-outcomes outside the forest environment, rather, they assigned the forest site with different condition as controls or did not assign controls among forest settings. Most of the included studies adopted cross-over designs to investigate the difference in the therapeutic effects in different forest settings. Only Bach et al. [85] and Elsadek et al. [86] assigned controls to urban sites, but their results were excluded with some reasons. Bach et al. [85] investigated blood levels of natural volatile organic compounds that have not been investigated in other studies. Elsadek et al. [86] conducted studies under high temperature conditions (28.1–31.8 °C) that are difficult to synthesize with other studies conducted under temperature conditions around 20 °C. We may regard one of the forest setting as a control, however, it will not be meaningful to combine urban settings and the forest settings as equivalent control groups. Thus, we estimated effect size by calculating a standardized mean difference of single groups. Accordingly, there would be a risk of resulting false positives and limitations in precision in deriving the effect size of forest therapy in this way. Future RCTs with control sites such as indoor or urban environments are required to derive more precise and reliable effect estimates. Nevertheless, the value of this study is verifying whether the therapeutic effect of the forest differs by structural characteristics of the forest environment. Thirdly, as we subgrouped the cases from included studies, sufficient sample size was required. However, the pooled populations were smaller than the appropriate sample sizes to examine the moderating effect on the alleviation of anxiety, depression, anger, and reinforcement of positive affect. With respect to canopy density, the collected samples were too small to confirm the moderating effects. Therefore, further studies on the therapeutic effects of forests in various canopy conditions are required.

The key challenge of this study was to investigate moderating role of stand density and canopy density. Through this study, we found that stand density modifies the level of therapeutic effects in the forest. Our findings had a great significance in suggesting the differences in therapeutic effects according to the environmental characteristics of nature. However, within the scope of this study, we cannot elucidate the underlying mechanisms. Several previous studies exploring differences in preference or beauty according to the characteristics of forest settings explained the differences using semantic difference measures [21,45,50,57,70] or physical-psychological predictors [74,75,76,77]. Especially, psychological mediators such as a sense of safety, visual access, and ease of movement were used to explain the difference in preference among forest settings with respect to structural variables [74,75,76]. The actual reason or psychological mechanisms are still under discussion [101]. Thus, there is abundant space for further progress in analyzing underlying mechanism that explains why forest sites with less stand density has more positive effects.

In addition, we found an intriguing trend as a result of additional analysis. There was a difference in the distribution of the therapeutic effect by region and forest management. The therapeutic effect of the forest reserve was the highest in all regions, and the order of the therapeutic effect of an urban forest and commercial forest differed by region. Future investigation on the impact of management type, landscape characteristics by country, and public use pattern are required.

## 5. Conclusions

In this study, a systematic literature review and meta-analysis were conducted to evaluate the relationship between the structural characteristics of forests and the therapeutic effect. We systematically searched, selected, and statistically synthesized the literature examining the therapeutic effects of forests with different stand density and canopy density. Subgroup analysis showed that stand density modifies the therapeutic effects. Emotional and cognitive restoration showed the largest effect size in low-density forests with a stand density of less than 500/ha and the therapeutic effects diminished as the stand density increases. The impact of canopy density has not been identified due to a lack of studies reporting canopy density. In addition, there would be a risk of false positives as we did not control the experimental design and control group. Although some limitations, we expect that our findings that highlight the impact of forest structure on the therapeutic effects of forests will contribute to advances in the therapeutic use of forests. We also expect that our findings may be used as a basis for creating adequate forest sites for future forest therapy.

## Figures and Tables

**Table 1:** Eligibility Criteria for Study Selection.

PICO	Inclusion Criteria	Exclusion Criteria
Participants	Humans who are healthy or not	Studies not including human participants
Intervention	Activities Spending time in the forest or urban forest (e.g., forest walking, forest viewing, forest bathing, forest therapy) Undertaken area Studies should describe forest environment in terms of stand density or canopy density (e.g., stand density, tree density, trees/ha, basal area, canopy density, canopy openness, canopy closure, sky view factor, etc.)	Studies with the intervention that does not match with defined activities Studies not reporting stand density nor canopy density
Comparator	Studies with a control site or not	NA
Outcome	Studies should report quantitative outcomes related to cognitive restoration, psychological restoration, physiological relaxation, stress reduction.	Studies not including quantitative outcomes

**Table 2:** Search Keywords.

PICO	Keywords
Intervention Activities	“forest therapy” OR “forest bathing” OR “shinrin-yoku” OR “shinrin yoku” OR “forest walk” OR “forest walking” OR “forest recreation” OR “nature therapy” OR “forest trip” OR “forest visit” OR (“trip” NEAR “forest”) OR (“visit” NEAR “forest”) OR (“spending time” NEAR “forest”) OR (“walking” NEAR “forest”) OR (“viewing” NEAR “forest”)
Undertaken Area	“stand density” OR “tree density” OR “strains/ha” OR “stands/ha” OR “trees/ha” OR “basal area” OR “canopy density” OR “canopy openness” OR “canopy closure” OR “sky view factor” OR “gap light analysis” OR (“managed” NEAR “forest”) OR (“unmanaged” NEAR “forest”) OR (“thinned” NEAR “forest”) OR (“unthinned” NEAR “forest”) OR “thinned condition” OR “unthinned condition” OR “thinning intensity” OR (“light environment” NEAR “tree vegetation”) OR (“light environment” NEAR “trees”) OR (“environmental factor” NEAR “forest”) OR (“environmental factor” NEAR “tree vegetation”) OR (“environmental factor” NEAR “trees”) OR (“microclimate” NEAR “forest”) OR (“microclimate” NEAR “tree vegetation”) OR (“microclimate” NEAR “trees”) OR (“physical environment” NEAR “forest”) OR (“physical environment” NEAR “tree vegetation”) OR (“physical environment” NEAR “trees”) OR (“forest” NEAR “structure”) OR “forest landscapes” OR “forest sites” OR “forests”
Outcome	“health” OR “well-being” OR “well-being” OR “psychological” OR “physiological” OR “psycho-physiological” OR “restorativeness” OR “restorative effect” OR “restorative experience” OR “psychological restoration” OR “PRS” OR “perceived restorativeness schedule” OR “ROS” OR “restorative outcome scale” OR “mood” OR “affective state” OR “POMS” OR “profile and mood state questionnaire” OR “PANAS” OR “positive and negative affect schedule” OR “anxiety” OR “STAI” OR “Spielberger state-trait anxiety inventory” OR “self-reporting anxiety scale” OR “depression” OR “BDI” OR “Beck depression inventory” OR “self-reporting depression scale” OR “vitality” OR “SVS” OR “subjective vitality scale” OR “psychological relaxation” OR “physiological relaxation” OR “psycho-physiological relaxation” OR “psychological response” OR “physiological response” OR “blood pressure” OR “SBP” OR “systolic blood pressure” OR “DBP” OR “diastolic blood pressure” OR “pulse rate” OR “heart rate” OR “HRV” OR “heart rate variability” OR “SDNN” OR “RMSSD” OR “LF” OR “HF” OR “LF/HF” OR “skin conductance” OR “SCR” OR “brain wave” OR “prefrontal activity” OR “SpO2” OR “EEG” OR stress reduction” OR “stress recovery” OR “stress restoration” OR “cortisol” OR “saliva”

**Table 3:** Characteristics of included studies.

**First** **Author and Year**	**Sample Size** **(M/F)**	**Participant** **Characteristics**	**Stand Density** **(Trees/ha)**	**DBH** **(cm)**		**Canopy Density**		**Forest** **Characteristics**	**Environmental** **Characteristics**		**Outcome** **Measurement**		**Activity Type**		**Time of Measure**	**Study** **Design**
An 2019 [84]	13 (7/6)	Healthy university students Age: 21.8 ± 1.9	3 different forest sites (no control assigned)					*Location:* 43°51′ N, 125°18′ E	*Temp:* 18 °C (10–25 °C) *Relative humidity:* 45% *Wind speed*: 3.33 m/s		SBP; DBP; HR		Staying (30 min)		Sep, 2017 (8:30–12:00)	Randomized Cross-over
			1667/ha	13.26		0.56		*Betula platyphylla*	*Illuminance*: 4617 lx		SBP(/); DBP(/); HR(/)					
			1867/ha	10.35		0.75		*Acer triflorum*	*Illuminance*: 1124 lx		SBP(/); DBP(/); HR(+)					
			1993/ha	14.39		0.78		*Quercus mongolica*	*Illuminance*: 1012 lx		SBP(+/); DBP(+/); HR(+/)					
Bach 2021 [85]	10 (6/4)	Healthy university students with no abnormalities in the respiratory or immune system Age: 36.4 ± 6.5	One forest site and urban site (control)					*Location:* 41°73′ N, 2°44′ E *Altitude:* 860–972 m *Dominant species:* *Quercus ilex*	*Temp:* 23.87 ± 0.18 °C *Relative humidity:* 53.7%		Blood concentration of Alpha-pinene(/); Beta-pinene(/); Alpha-phellandrene(/); Limonene(/); All monoterpene(/)		Walking (120 min)		July, 2018 (10:00–12:00)	Randomized Controlled Trial
			10500/ha	6.43		0.95										
Bielinis 2019 [27]	21 (12/9)	Healthy university students and non-student volunteers with no mental or physical diseases or metabolic syndromes Age: 23.9 ± 2.7	One forest group (no control)					*Location:* 53°90′ N, 20°35′ E *Dominant species:* *Picea abies* and *Pinus sylvestris*	*Temp:* 25 °C *Relative humidity:* 46% *Wind speed*: 6.11 m/s *Noise:* 38.08 ± 5.19 dB *Illuminance:* 37755.24 lx		SBP(+); DBP(/); PR(+); MAP(+); POMS(Confusion(+) Fatigue(+/) Anger(+) Anxiety(+) Depression(+) Vigor(/)); PANAS(PA(/) NA(+)); ROS(+); SVS(+)		Staying (60 min)		May, 2018 (15:45–16:15)	Uncontrolled Before and After
			1200/ha	NA		NA										
Elsadek 2019 [86]	346 (200/164)	Healthy university students with no mental or physical diseases Age: 23.0 ± 4.6	3 different forest sites and urban site(control)					Location: 31°27′ N, 121°46′ E			POMS; STAI; ROS; SVS; PET		Walking (15 min)		May, 2018 (10:00–15:30)	Randomized Crossover
			NA	NA		0.13		Urban road (control)	Temp: 31.8 °C Relative humidity: 42.8% Wind speed: 0.66 m/s		POMS(Anxiety(−) Depression(−) Anger(−) Fatigue(−) Confusion(−) Vigor(−)); STAI(−); ROS(−); SVS(−)					
						0.49		*Prunus serrulata*	Temp: 31.2 °C Relative humidity: 47.0% Wind speed: 0.23 m/s		POMS(Anxiety(+) Depression(+) Anger(+) Fatigue(+) Confusion(+) Vigor(+)); STAI(+); ROS(+); SVS(+)					
						0.89		*Plantanus hispanica*	Temp: 29.0 °C Relative humidity: 50.9% Wind speed: 0.15m/s		POMS(Anxiety(+) Depression(+) Anger(+) Fatigue(+) Confusion(+) Vigor(+)); STAI(+); ROS(+); SVS(+)					
						0.94		*Metasequoia glyptroboides*	Temp: 28.1 °C Relative humidity: 52.9% Wind speed: 0.11 m/s		POMS(Anxiety(+) Depression(+) Anger(+) Fatigue(+) Confusion(+) Vigor(+)); STAI(+); ROS(+); SVS(+)					
Lee 2020 [87]	16 (5/11)	Housewives, freelancers and office workers Age: 41.6 ± 1.8	One forest site and one outdoor site(control)					Location*:* 35°83′ N, 128°76′ E Dominant species: *Pinus rigida* and *Quercus aliena*	NA		DEP(+/); ANX(+/); AGG(/); ST-IN-S(+); ST-V-RG-P(+)		Staying (120 min)		May–Aug, 2017	Controlled Before and After
			156/ha	27.4		NA										
Oishi 2003 [88]	44 (23/21)	Healthy residents including students and workers Age: 24.4 ± 9.9	5 different forest sites and one site outside the forest(control)					Location: 39${}^{\circ}75^{\prime }$ N, 141°03′ E			POMS		Staying (10 min)	Jul–Sep, 2001 (8:45–13:10)		Non-Randomized Cross-over
			250/ha	23		NA		*Pinus densiflora*	Temp: 19.67 ± 3.31 °C Relative humidity: 68.9%		POMS(Anxiety(+) Depression(+) Anger(+) Fatigue(+) Confusion(+) Vigor(+))					
			750/ha	34.9				*Cyptomeria japonica*	Temp: 20.29 ± 3.66 °C Relative humidity: 69.3%		POMS(Anxiety(+) Depression(+) Anger(+) Fatigue(+) Confusion(+) Vigor(+))					
			1700/ha	10.6				*Quercus serrata*	Temp: 19.91 ± 3.40 °C Relative humidity: 75.8%		POMS(Anxiety(+) Depression(+) Anger(+) Fatigue(+) Confusion(+) Vigor(+))					
			2275/ha	10.4				*Pinus densiflora* *Machilus thunbergii*	Temp: 20.17 ± 3.19 °C Relative humidity: 73.3%		POMS(Anxiety(+) Depression(+) Anger(+) Fatigue(+) Confusion(+) Vigor(+))					
			2875/ha	9.5				*Pinus densiflora* *Quercus serrata*	Temp: 18.63 ± 3.16 °C Relative humidity: 76.4%		POMS(Anxiety(+) Depression(+) Anger(+) Fatigue(+) Confusion(+) Vigor(+))					
Saito 2019 [89]	17 (17/-)	healthy male volunteer had no history of cardiovascular disease or mental illness Age: 40.2 ± 6.2	2 different forest sites: managed forest and unmanaged forest(control)					Location: 35°41′ N, 138°86′ E	Wind speed: m/s		SBP; DBP; MAP; HR; HF; LF/HF; saliva cortisol		Staying (15 min)	July, 2014		Randomized Crossover
			1024/ha	19.78		0.62		Mixed forest (*Broadleaf trees*: 82% *Coniferous trees:* 18%)	Temp: 24.3 ± 2.8 °C Relative humidity: 73.3% Illuminance: 255.0 ± 97.6 lx		SBP(+); DBP(+); MAP(+); HR(/); HF(+/); LF/HF(+); saliva cortisol(+)					
			1208/ha	15.75		0.91		Mixed forest (*Broadleaf trees*: 50% *Coniferous trees:* 50%)	Temp: 24.2 ± 2.8 °C Relative humidity: 70.0% Illuminance: 119.2 ± 48.5 lx		SBP(+); DBP(+); MAP(+); HR; HF(−/); LF/HF(−); saliva cortisol(+/)					
Simkin 2020 [90]	66 (41/59)	City workers aged from 26–65 Age: 43.4 ± 10.7	4 different forest sites (no control assigned)					*Picea abies-*dominated forests in Finland			ROS; SVS; PANAS		Staying (15 min) and Walking (45 min)	Aug–Oct, 2016 April–June, 2017 (15:00–17:00)		Randomized Crossover
			374/ha	35		NA		Location: 60°45′ N, 25°19′ E (stand age > 120)	Temp: 12.8 ± 4.4 °C Relative humidity: 39–96%		ROS(+); SVS(+); PANAS(+)					
			424/ha	30				Location: 60°22′ N, 24°92′ E (stand age: 95)	Temp: 15.8 ± 4.2 °C Relative humidity: 29–100%		ROS(+); SVS(+); PANAS(+)					
			520/ha	28				Location: 60°33′ N, 25°18′ E (stand age: 100)	Temp: 15.9 ± 5.8 °C Relative humidity: 39–91%		ROS(+); SVS(+); PANAS(+)					
			1746/ha	16				Location: 60°39′ N, 25°18′ E (stand age: 40)	Temp: 15.3 ± 4.8 °C Relative humidity: 27–98%		ROS(+); SVS(+); PANAS(+)					
Takayama 2017a [91]	18 (18/-)	Healthy male volunteer without history of cardiovascular disease or mental illness Age: 40.2 ± 6.4	2 different forest sites: managed forest and unmanaged forest(control)					Location: 35°41′ N, 138°86′ E			POMS; PANAS; ROS; PRS		Staying (15 min)	July, 2014		Randomized Crossover
			1056/ha	23.12		0.62		*Larix kaempferi* 66%, *Cornus controversa* 10% *Pinus densiflora* 7%	Temp: 24.3 ± 7.9 °C Relative humidity: 73 ± 39% Wind velocity: 0.13 m/s Radiant Heat: 25.5 ± 10.5 °C Illuminance: 255.0 ± 97.6 lx Noise: 41.5 ± 3.8 dB		POMS(Anxiety(+) Depression(/) Anger(/) Fatigue(+/) Confusion(+) Vigor(+)); PANAS(PA(/)NA(+)); ROS(+)					
			1212/ha	18.54		0.91		*Larix kaempferi* 66% *Cornus controversa* 7% *Pinus densiflora* 7%	Temp: 24.2 ± 7.6 °C Relative humidity: 70 ± 34% Wind velocity: 0.23 m/s Radiant Heat: 25.3 ± 9.8 °C Illuminance: 119.2 ± 48.5 lx Noise: 39.0 ± 3.9 dB		POMS(Anxiety(+) Depression(/) Anger(/) Fatigue(/) Confusion(+) Vigor(+)); PANAS(PA(/)NA(+/)) ROS(/)					
Takayama 2017 [92]	15 (11/4)	University students and mid-aged faculties without had no history of cardiovascular disease or mental illness Age: 35.9 ± 8.2	2 different forest sites: thinned forest and unthinned forest(control)					Location: 35°41′ N, 138°86′ E		POMS; PANAS; ROS; PRS		Staying (15 min)		Oct, May, 2013		Randomized Crossover
			1056/ha	23.12		0.52		*Larix kaempferi* 66%, *Cornus controversa* 10% *Pinus densiflora* 7%	Temp: 18.6 ± 0.92 °C Relative humidity: 92 ± 4.6% Wind velocity: 0.16 m/s Radiant Heat: 18.8 ± 1.1 °C Illuminance: 336 ± 172 lx	POMS(Anxiety(+) Depression(+) Anger(+) Fatigue(+/) Confusion(+) Vigor(/)); PANAS(PA(/)NA(/)) ROS(+/)						
			1200/ha	22.76		0.59		*Larix kaempferi* 66%, *Cornus controversa* 10% *Pinus densiflora* 7%	Temp: 17.1 ± 2.52 °C Relative humidity: 38 ± 20% Wind velocity: 0.30 m/s Radiant Heat: 21.8 ± 2.8 °C Illuminance: 668 ± 237 lx	POMS(Anxiety(+) Depression(+) Anger(/) Fatigue(+) Confusion(+) Vigor(/)); PANAS(PA(/)NA(/)) ROS(+)						
Yu [93]	123 (43/85)	Middle-aged and elderly subjects aged 45 to 86 years No disease (*n* = 69), hypertension (*n* = 25), diabetes (*n* = 9), heart disease (*n* = 8) Age: 60.0 ± 7.4	One forest site (no control)					Location: 24°09′ N, 121°18′ E Dominant species: *Cryptomeria japonica*	Temp: 22.6 ± 1.4 °C Relative humidity: 87.4% Wind speed: 0.1 ± 0.2 m/s	PR(+); SBP(+); DBP(+); HF(/); LF/HF(/); POMS(Anxiety(+) Depression(+) Anger(+) Fatigue(+) Confusion(+) Vigor(+)); STAI(+)		Walking (180 min)		July, 2016 (8:30–12:00)		Uncontrolled Before and After
			1200/ha	NA		NA										
Zhou 2019 [94]	43 (8/35)	University students free from diagnosed cardiovascular, allergic, physical disease, or mental diseases Age: 20.8 ± 0.9	2 different forest sites (no control assigned)					Location: 26°11′ N–26°55′ N 106°07′ E–107°17′ E	Temp: 22.6 ± 1.4 °C Relative humidity: 87.4% Wind speed: 0.1 ± 0.2 m/s		Anti-anxiety scores	walking (60 min)		Dec, 2016		Randomized Cross-over
			NA		NA		0.20–0.75	*Cinnamomum camphora* *Quercus fabri* *Platanus acerifolia*			Anti-anxiety scores (financial states(+); exam pressure(+); campus life(/); love affair relationship(+))					
							0.50–0.75	*Cinnamomum camphora* *Celtis sinensis* *Catalpa bungei* *Celtis sinensis*			Anti-anxiety scores(financial states(+); exam pressure(+); campus life(+); love affair relationship(/))					

*AGG* 7—questions on aggression, *ANX* 11—questions on anxiety, *DBP*—diastolic blood pressure, *DEP* 11—questions on depression, *HBP—*high-frequency, *HR—*heart rate, *LF/HF—*ratio of low-frequency and high frequency, *MAP—*mean arterial pressure, *PANAS—*positive and negative affect schedule, *POMS—*profile of mood states, *PR—*pulse rate, *PRS—*perceived restorativeness scale, *ROS—*restorative outcomes scale, *SBP*—systolic blood pressure, *STAI—*state trait anxiety inventory, *ST-V-IN-S—*five questions on stress vulnerability-interpersonal sensitivity, *ST-V-RG-P—*five questions on stress vulnerability-self-regulation problems, *SVS—*subjective vitality scale, *Temp*—temperature. +: significant effect on positive health outcome; +/: including both significant and nonsignificant effect on positive health outcome; /: nonsignificant effect; -/: including both significant and nonsignificant effect on negative health outcome -: significant effect on negative outcome.

**Table 4:** Methodological Quality Assessment of Included Studies Using ROBINS-I.

Study		Pre-Intervention		At Intervention	Post-Intervention				Overall Risk of Bias
First Author	Year	Bias Due to Confounding	Bias in Selection of Participants into the Study	Bias in Classification of Interventions	Bias Due to Deviations from Intended Interventions	Bias Due to Missing Data	Bias in Measurement of Outcomes	Bias in Selection of the Reported Result	
An	2019	Low	Low	Low	Low	Low	Low	Moderate	Moderate
Bach	2021	Low	Low	Low	Low	Low	Low	Low	Low
Bielinis	2019	Low	Moderate	Low	Low	Low	Moderate	Moderate	Moderate
Elsadek	2019	Low	Moderate	Low	No Information	No Information	Moderate	Moderate	No information
Lee	2020	Serious	Moderate	Low	Low	Low	Moderate	Moderate	Serious
Oishi	2003	Serious	Moderate	Low	Low	Low	Moderate	Moderate	Serious
Saito	2019	Low	Low	Low	Low	Low	Low	Moderate	Moderate
Simkin	2020	Low	Low	Low	Low	Low	Moderate	Moderate	Moderate
Takayama	2017a	Low	Low	Low	Low	Low	Moderate	Serious	Serious
Takayama	2017b	Low	Low	Low	Low	Low	Moderate	Moderate	Moderate
Yu	2017	Low	Moderate	Low	Low	Low	Moderate	Moderate	Moderate
Zhou	2019	Low	Low	Low	Low	Low	Moderate	Moderate	Moderate

## Supplementary Materials

The following are available online at https://www.mdpi.com/article/10.3390/healthcare9111427/s1, Table S1: PRISMA 2020 checklist; Figure S1: result of sensitivity analysis.
