# Supplementary material for: Can Different Forest Structures Lead to Different Levels of Therapeutic Effects? A Systematic Review and Meta-Analysis
Source: Healthcare (Basel). 2021 Oct 23;9(11):1427. doi: 10.3390/healthcare9111427 (PMC8623963; doi:10.3390/healthcare9111427)

Figure S1. Result of Sensitivity Analysis

3.3.1. Emotional Restoration

Anxiety

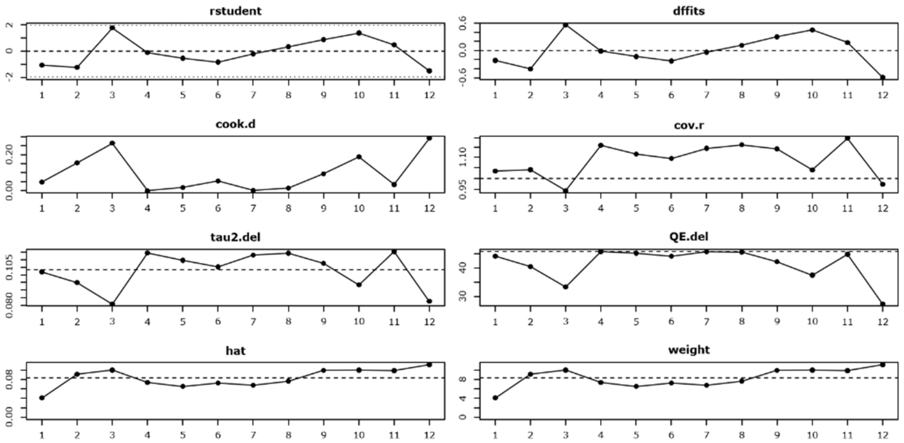

Depression

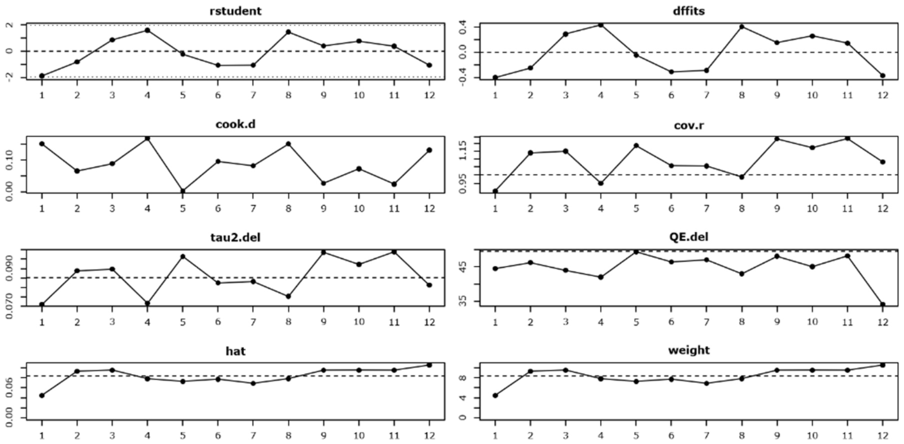

Anger

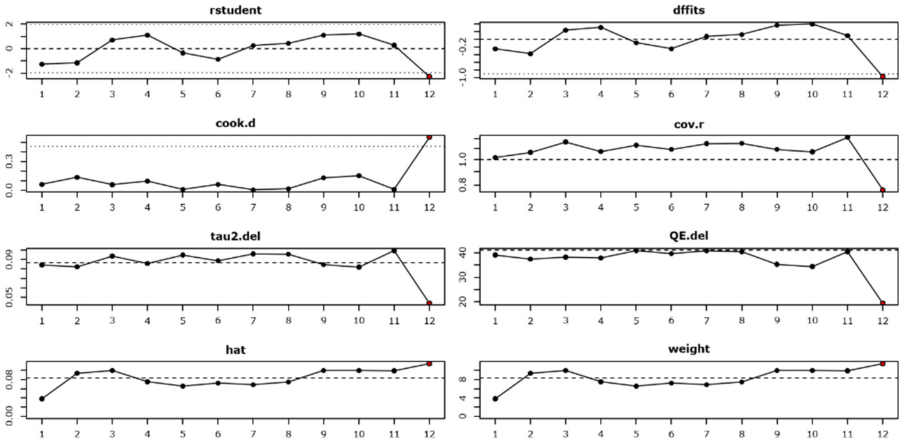

Fatigue

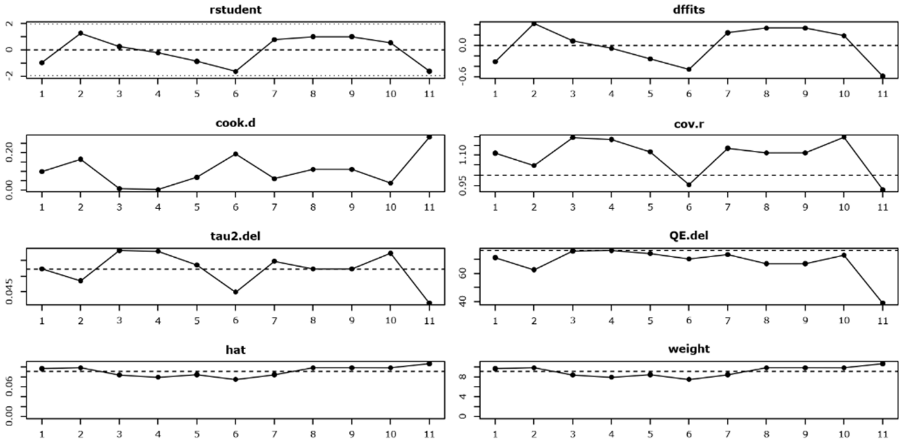

Vigor

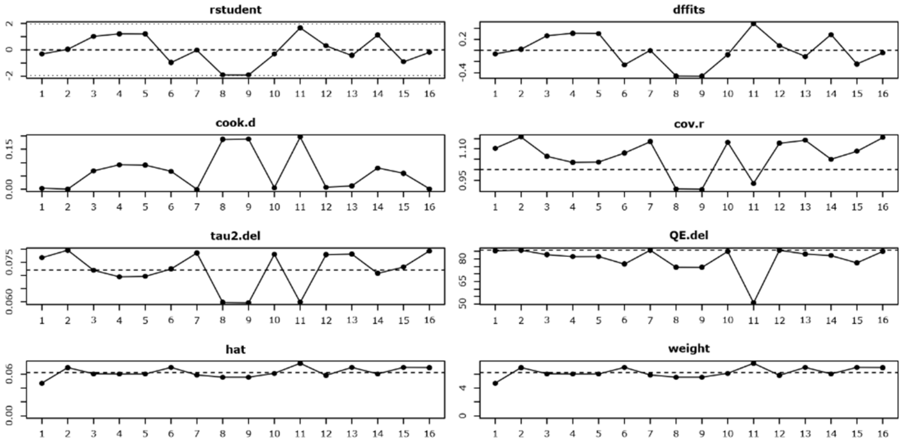

Negative Affect

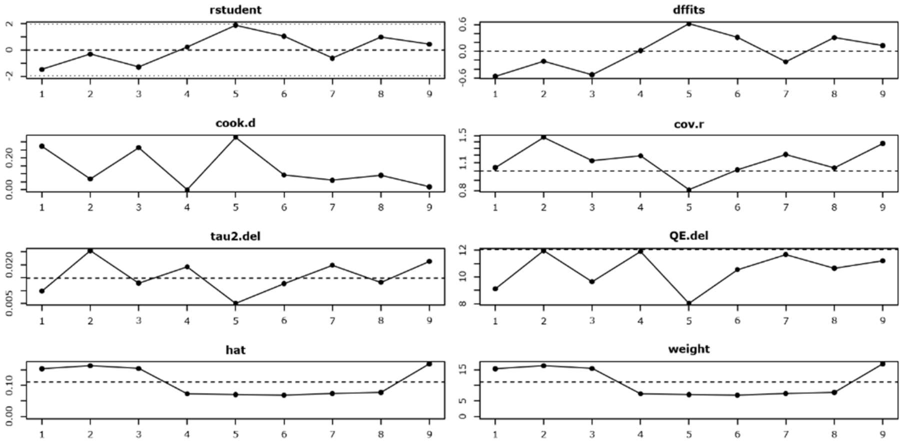

Positive Affect

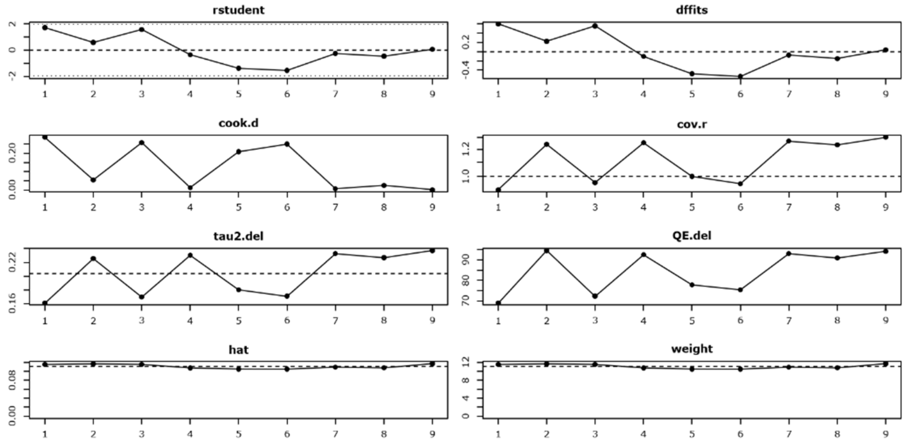

3.3.2. Cognitive Restoration

Restorative experience

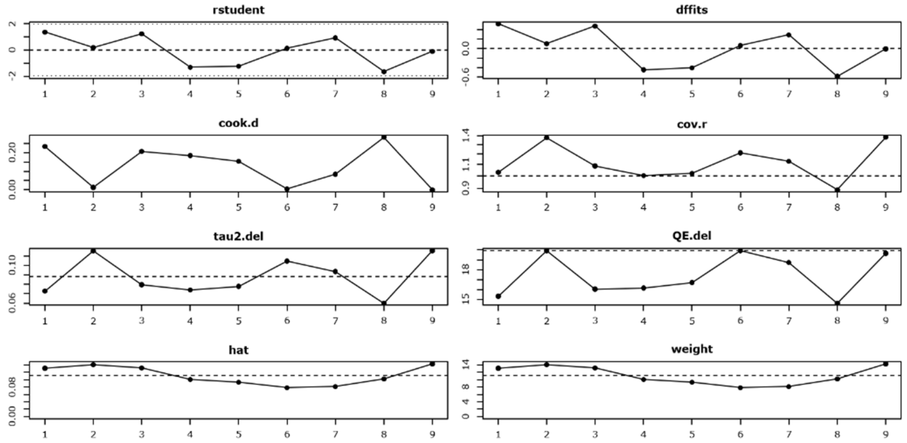

3.3.3. Physiological Relaxation

Diastolic Blood Pressure

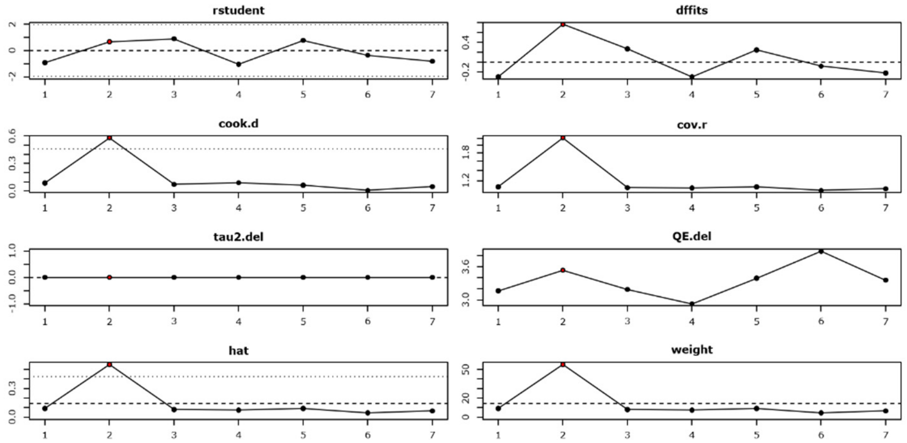

Systolic Blood Pressure

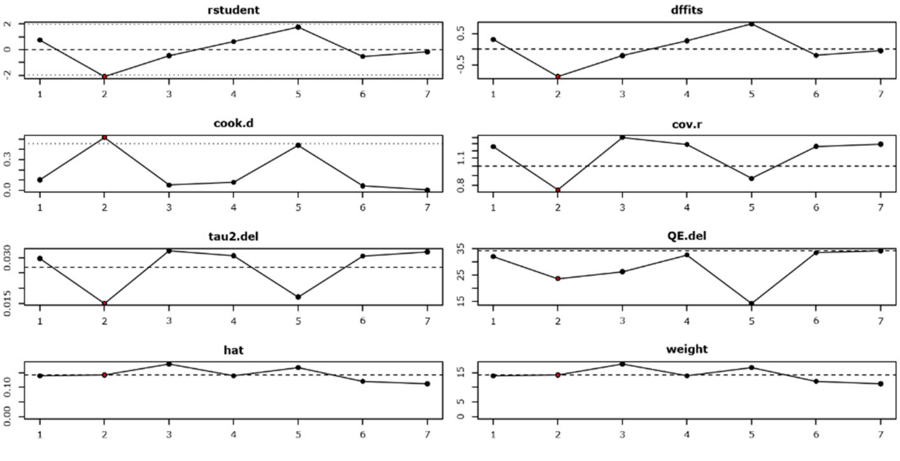

Heart Rate or Pulse Rate

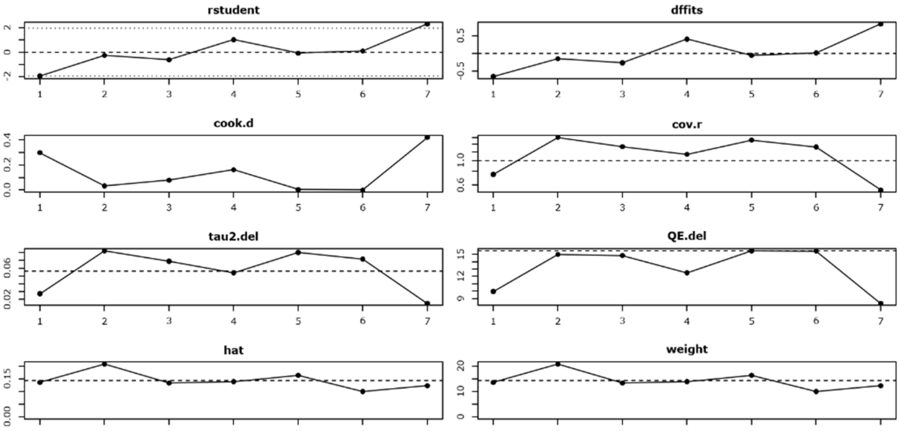

3.4.1. Physiological Relaxation

Diastolic Blood Pressure

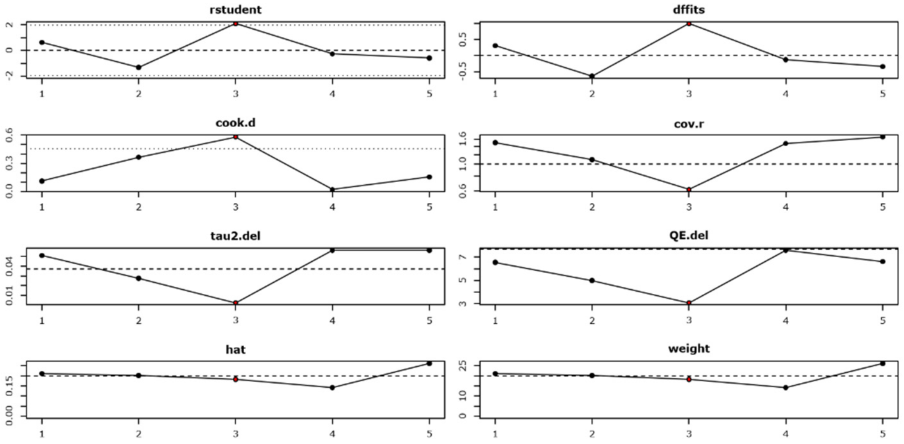

Systolic Blood Pressure

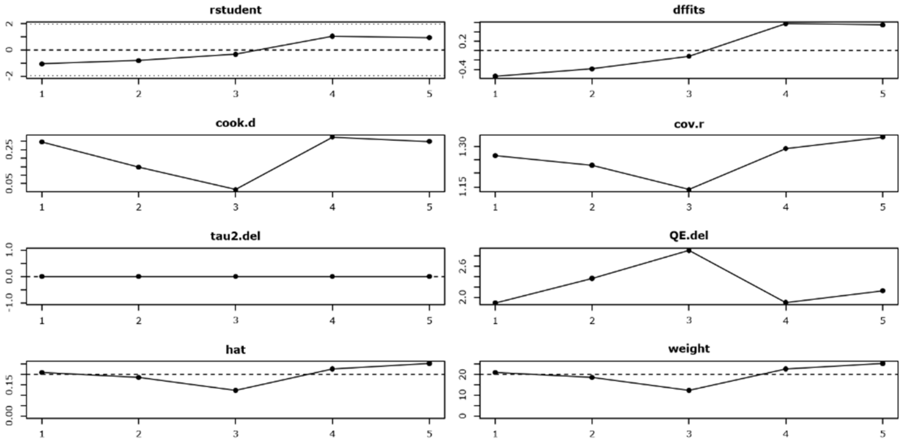

Heart Rate or Pulse Rate

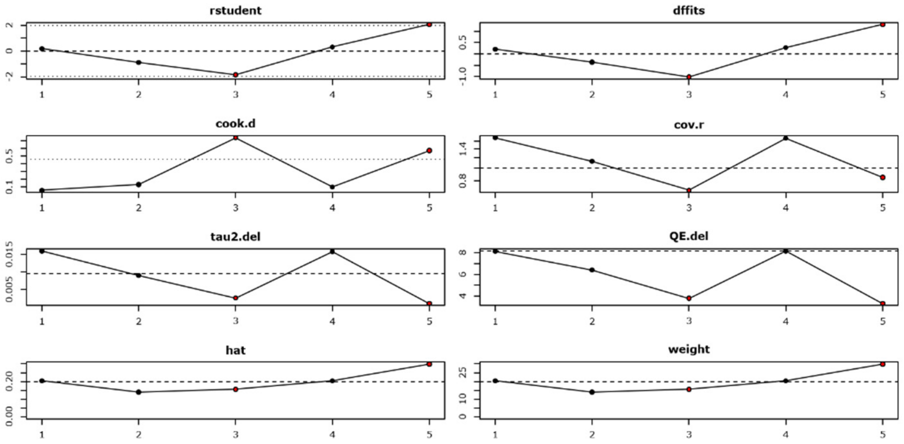

Supplement: Supplementary file 1 [file healthcare-09-01427-s001.zip › Figure S1_Result of Sensitivity Analysis.pdf]
